# Efficacy and Safety of Stapokibart in Adults With Moderate‐to‐Severe Atopic Dermatitis With and Without Type 2 Comorbidities: A Post Hoc Analysis of a Phase 3 Trial

**DOI:** 10.1002/clt2.70121

**Published:** 2025-11-19

**Authors:** Yan Zhao, Litao Zhang, Liming Wu, Bin Yang, Jinyan Wang, Yumei Li, Qingchun Diao, Jingyi Li, Qing Sun, Xiaohong Zhu, Xiaoyong Man, Lihua Wang, Yanyan Feng, Tao Cai, Huiming Zeng, Linfeng Li, Jianyun Lu, Hong Ren, Fuqiu Li, Qianjin Lu, Xiaohua Tao, Rong Xiao, Chao Ji, Wenjie Zhao, Wei Chu, Bo Chen, Jianzhong Zhang

**Affiliations:** ^1^ Department of Dermatology Peking University People's Hospital Beijing China; ^2^ Department of Dermatology Tianjin Academy of Traditional Chinese Medicine Affiliated Hospital Tianjin China; ^3^ Department of Dermatology Affiliated Hangzhou First People's Hospital School of Medicine Westlake University Hangzhou Zhejiang China; ^4^ Department of Dermatology Dermatology Hospital Southern Medical University Guangzhou Guangdong China; ^5^ Department of Dermatology Ningbo No. 2 Hospital Ningbo Zhejiang China; ^6^ Department of Dermatology Affiliated Hospital of Jiangsu University Zhenjiang Jiangsu China; ^7^ Department of Dermatology Chongqing Traditional Chinese Medicine Hospital Chongqing China; ^8^ Department of Dermatovenereology West China Hospital of Sichuan University Chengdu Sichuan China; ^9^ Department of Dermatology Qilu Hospital of Shandong University Jinan Shandong China; ^10^ Department of Dermatology Wuxi No.2 People's Hospital (Jiangnan University Medical Center) Wuxi Jiangsu China; ^11^ Department of Dermatology Second Affiliated Hospital Zhejiang University School of Medicine Hangzhou Zhejiang China; ^12^ Department of Dermatology Central Hospital Affiliated to Shandong First Medical University Jinan Shandong China; ^13^ Department of Dermatology Chengdu Second People's Hospital Chengdu Sichuan China; ^14^ Department of Dermatology The First Affiliated Hospital of Chongqing Medical University Chongqing China; ^15^ Department of Dermatology The First Affiliated Hospital of Hainan Medical University Haikou Hainan China; ^16^ Department of Dermatology Beijing Friendship Hospital Capital Medical University Beijing China; ^17^ Department of Dermatology The Third Xiangya Hospital of Central South University Changsha Hunan China; ^18^ Department of Dermatology The First People's Hospital of Lianyungang Lianyungang Jiangsu China; ^19^ Department of Dermatology The Second Hospital of Jilin University Changchun Jilin China; ^20^ Hospital for Skin Diseases Institute of Dermatology Chinese Academy of Medical Sciences and Peking Union Medical College Nanjing Jiangsu China; ^21^ Key Laboratory of Basic and Translational Research on Immune‐Mediated Skin Diseases Chinese Academy of Medical Sciences Nanjing Jiangsu China; ^22^ Jiangsu Key Laboratory of Molecular Biology for Skin Diseases and STIs Nanjing Jiangsu China; ^23^ Center for Plastic & Reconstructive Surgery Department of Dermatology Zhejiang Provincial People's Hospital (Affiliated People's Hospital, Hangzhou Medical College) Hangzhou Zhejiang China; ^24^ Department of Dermatology and Venereology The Second Xiangya Hospital of Central South University Changsha Hunan China; ^25^ Department of Dermatology The First Affiliated Hospital of Fujian Medical University Fuzhou Fujian China; ^26^ Keymed Biosciences (Chengdu) Co. Ltd Chengdu Sichuan China

**Keywords:** atopic dermatitis, stapokibart, type 2 comorbidities

## Abstract

**Background:**

Atopic dermatitis (AD) often coexists with other type 2 inflammatory diseases. Stapokibart, a humanized IgG4 monoclonal antibody targeting interleukin‐4 receptor alpha subunit, showed high efficacy and favorable safety in a phase 3 trial. This post‐hoc analysis aimed to compare the efficacy and safety of stapokibart in AD patients with and without type 2 comorbidities.

**Methods:**

During 16‐week double‐blind period, participants were randomly assigned to stapokibart 600 (loading dose)‐300 mg (*n* = 251) or placebo (*n* = 249) treatment every other week (Q2W). All patients received stapokibart 300 mg Q2W during subsequent 36‐week maintenance period. Patients with ≥ 1 of the following conditions were classified into comorbid subgroup: allergic rhinitis, asthma, food allergies, chronic urticaria, or chronic obstructive pulmonary disease. Post‐hoc outcomes included response rates of ≥ 75%/90% improvement in Eczema Area and Severity Index score (EASI‐75/90), Investigator's Global Assessment score of 0 or 1 (IGA 0/1) with ≥ 2‐point reduction, and ≥ 4‐point reduction in weekly average of daily peak pruritus numerical rating scale score (PP‐NRS4), and the percentage change from baseline in weekly average of daily PP‐NRS score. Treatment‐emergent adverse events (TEAEs) were also analyzed by subgroups.

**Results:**

Ninety‐two patients (36.7%) in stapokibart group and 102 (41.0%) in placebo group had type 2 comorbidities. The demographic characteristics and baseline disease scores were generally comparable across four groups. At week 16, stapokibart demonstrated superior efficacy over placebo in patients with and without type 2 comorbidities. In patients with comorbidities, the response rates of EASI‐75, IGA 0/1, and PP‐NRS4 in stapokibart and placebo groups were 77.2% versus 26.5%, 55.4% versus 17.6%, and 43.5% versus 12.7%, respectively. In patients without comorbidities, these response rates were 61.0% versus 25.3%, 37.7% versus 15.1%, and 31.4% versus 11.0%, respectively (all *p* values < 0.0001). All patients showed further improvements in efficacy outcomes during weeks 20–52. TEAEs occurred in 88.8% and 87.7% of comorbid and non‐comorbid patients over weeks 0–52. The incidence of conjunctivitis was 5.9% and 5.0%, respectively.

**Conclusion:**

Stapokibart was effective and safe in adults with moderate‐to‐severe AD both with and without type 2 comorbidities in both short‐term and long‐term treatment.

**Trial Registration:**

ClinicalTrials.gov identifier: NCT05265923

## 1 Introduction

Atopic dermatitis (AD), characterized by chronic eczematous skin lesions and pruritus, often greatly diminishes patients' quality of life [1]. AD exhibits shared pathogenesis with other atopic disorders, primarily through dysregulated type 2 inflammation. This process involves hyperactivation of T‐helper type 2 (Th2) cells and innate lymphoid cells, stimulating the production of key cytokines like interleukin (IL)‐4, IL‐5, and IL‐13 [2]. Therefore, allergic rhinitis (AR), asthma, food allergies (FA), chronic rhinitis with nasal polyps (CRSwNP), and other type 2 inflammatory disorders, are common comorbidities of AD [3, 4, 5]. Moreover, AD is associated with an increased risk of developing other atopic disorders [5, 6], known as atopic march or atopic clustering, and the likelihood of type 2 comorbidities (particularly asthma and AR) correlates positively with AD severity [7]. The prevalences of AR and asthma were 29.3% and 25.7% among patients with AD, compared to 18.0% and 8.1% among reference individuals without AD, respectively, in systematic review and meta‐analyses [8, 9].

Although conventional topical medications (e.g., corticosteroids and calcineurin inhibitors) can effectively relieve AD symptoms, patients with moderate‐to‐severe AD (msAD) report suboptimal efficacy and low treatment satisfaction [10, 11]. On the other hand, long‐term treatment with systemic immunosuppressants and systemic corticosteroids should be avoided by potentially severe side effects [12]. This highlights the needs for new treatment options for msAD with satisfactory clinical efficacy and tolerable toxicities. In addition, the presence of comorbidities may affect treatment selections. In patients with comorbid autoimmune diseases who have a heavier disease burden, newer treatments such as Janus kinase inhibitors and biologics may be preferred [3].

Stapokibart, a novel humanized IgG4 monoclonal antibody selectively binding IL‐4 receptor alpha subunit (IL‐4Rα), blocks the signaling of both IL‐4 and IL‐13. Preclinical studies demonstrated comparable or numerically superior blocking activity of stapokibart to IL‐4Rα compared with dupilumab [13]. In a phase 3 trial (CM310AD005), 16 weeks of stapokibart treatment led to response rates of ≥ 75% improvement from baseline in Eczema Area and Severity Index score (EASI‐75) and Investigator's Global Assessment score of 0 or 1 (IGA 0/1) of 66.9% and 44.2%, significantly higher than the corresponding rates with placebo (25.8% and 16.1%, respectively) [14]. The favorable treatment efficacy was maintained through week 52 with a manageable safety profile [15]. Stapokibart has received its approval from the National Medical Products Administration (NMPA) for treating adults with msAD [16].

This post‐hoc analysis evaluated the application of stapokibart across AD subpopulations stratified by type 2 comorbidity presence.

## 2 Methods

### 2.1 Study Design

CM310AD005 was a randomized, double‐blind, phase 3 trial involving adults with msAD conducted in 59 medical centers in China, and the details of the study design have been reported previously [14]. Eligible participants were randomized to receive 16‐week subcutaneous injections of stapokibart 600 (loading dose)‐300 mg or placebo at a 1:1 ratio every other week (Q2W). Subsequently, both groups received open‐label maintenance stapokibart treatment (300 mg Q2W) for 36 weeks. All patients used moisturizers twice daily during the entire study period, and topical AD therapies were allowed during the maintenance treatment period.

This trial adhered to the ethical standards of the Declaration of Helsinki and followed the Good Clinical Practice regulations established by the NMPA. The research protocol, including any modifications, received approval from the ethics committee of all involved sites. Written informed consent was obtained from each participant prior to any study procedure.

### 2.2 Outcomes

Efficacy outcomes, pharmacodynamic biomarkers, and safety outcomes were analyzed in subgroups with and without type 2 comorbidities. Patients with ≥ 1 type 2 comorbidity (AR, asthma, FA, chronic urticaria, or chronic obstructive pulmonary disease) at baseline comprised the comorbid subgroup. Efficacy outcomes included response rates of EASI‐75, EASI‐90, IGA 0/1 with ≥ 2‐point reduction, and ≥ 4‐point reduction in weekly average of daily peak pruritus numerical rating scale score (PP‐NRS4), and the percentage change from baseline in weekly average of daily PP‐NRS score. Pharmacodynamic biomarkers were assessed over weeks 0–16, including serum thymus and activation‐regulated chemokine (TARC), total IgE, and lactate dehydrogenase (LDH). Safety outcomes were treatment‐emergent adverse events (TEAEs).

### 2.3 Statistical Analysis

For response rates, non‐responder imputation (NRI) was applied to missing data during weeks 0–16, while data beyond week 16 were analyzed as observed. The 95% confidence intervals (CIs) of the between‐group differences of the response rates at week 16 were computed using the stratified Newcombe method (stratified by baseline IGA score, 3 vs. 4), with *p* values calculated by the Cochran‐Mantel‐Haenszel method. The percentage changes from baseline in weekly average of daily PP‐NRS score were analyzed based on observed data, using the mixed‐effect model for repeated measures (MMRM) during weeks 0–16 and descriptive statistics beyond week 16. Covariates for MMRM included treatment, visit, treatment‐by‐visit interaction, randomization strata (baseline IGA score of 3 vs. 4), baseline PP‐NRS score, and baseline‐by‐visit interaction. The treatment‐by‐subgroup interaction at week 16 was evaluated using the logistic regression model for binary outcomes and the analysis of covariance model for the continuous outcome. Statistical analysis was conducted using SAS version 9.4 (SAS Institute, Cary, NC).

## 3 Results

### 3.1 Baseline Characteristics and Concomitant Medications

In patients enrolled in CM310AD005, 36.7% (92/251) patients in stapokibart group and 41.0% (102/249) patients in placebo group had type 2 comorbidities. The demographic characteristics were generally similar, and the baseline disease scores, TARC level and LDH level were comparable between 4 subgroups. Serum total IgE level was higher in comorbid patients than non‐comorbid patients. The most common type 2 comorbidity was AR, followed by asthma (Table 1). During the maintenance treatment and follow‐up period (weeks 16–60), topical corticosteroids and topical calcineurin inhibitors were used in 24.5% and 14.7% patients with comorbidities, and in 21.5% and 12.4% patients without comorbidities, respectively (Supporting Information S1: Table S1).

**TABLE 1:** Baseline demographics and clinical characteristics in patients with and without type 2 comorbidities.

Characteristics	Comorbid		Non‐comorbid	
	Stapokibart (*n* = 92)	Placebo (*n* = 102)	Stapokibart (*n* = 159)	Placebo (*n* = 147)
Age (years)	36.1 ± 16.5	36.9 ± 15.5	42.7 ± 16.4	41.3 ± 17.1
Sex				
Male	55 (59.8)	62 (60.8)	104 (65.4)	97 (66.0)
Female	37 (40.2)	40 (39.2)	55 (34.6)	50 (34.0)
Body mass index (kg/m^2^)	22.55 ± 3.38	23.53 ± 4.04	24.20 ± 3.55	23.98 ± 3.42
Duration of AD (years)	7.0 (3.0–16.5)	7.5 (4.0–17.0)	7.0 (3.0–12.0)	5.0 (3.0–10.0)
EASI score	23.49 ± 8.20	25.38 ± 9.72	25.62 ± 8.88	23.13 ± 7.60
IGA score				
3	56 (60.9)	47 (46.1)	75 (47.2)	84 (57.1)
4	36 (39.1)	55 (53.9)	84 (52.8)	63 (42.9)
Weekly average of daily PP‐NRS score	7.23 ± 1.45	7.31 ± 1.57	7.26 ± 1.50	7.12 ± 1.63
Serum TARC (pg/mL)	478.0 (241.0–1085.0)	386.0 (209.0–900.0)	377.0 (195.0–821.0)	376.0 (192.0–763.0)
Serum total IgE (ng/mL)	1696.5 (407.2–6898.5)	1660.5 (267.8–10230.0)	639.4 (174.4–3148.0)	529.3 (135.7–2400.0)
LDH (U/L)	215.0 (179.0–268.0)	221.5 (187.6–262.0)	231.0 (194.0–280.0)	217.5 (188.0–266.0)
Type 2 comorbidities				
Allergic rhinitis	84 (91.3)	90 (88.2)	NA	NA
Asthma	15 (16.3)	21 (20.6)	NA	NA
Chronic obstructive pulmonary disease	1 (1.1)	1 (1.0)	NA	NA
Chronic urticaria	4 (4.3)	2 (2.0)	NA	NA
Food allergies	3 (3.3)	4 (3.9)	NA	NA

*Note:* Data are mean ± standard deviation, *n* (%), or median (interquartile range).

Abbreviations: AD, atopic dermatitis; EASI, eczema area and severity index; IGA, investigator's global assessment; LDH, lactate dehydrogenase; NA, not applicable; PP‐NRS, peak pruritus numerical rating scale; TARC, thymus and activation‐regulated chemokine.

### 3.2 Efficacy and Pharmacodynamic Outcomes During Weeks 0–16

**FIGURE 1:**
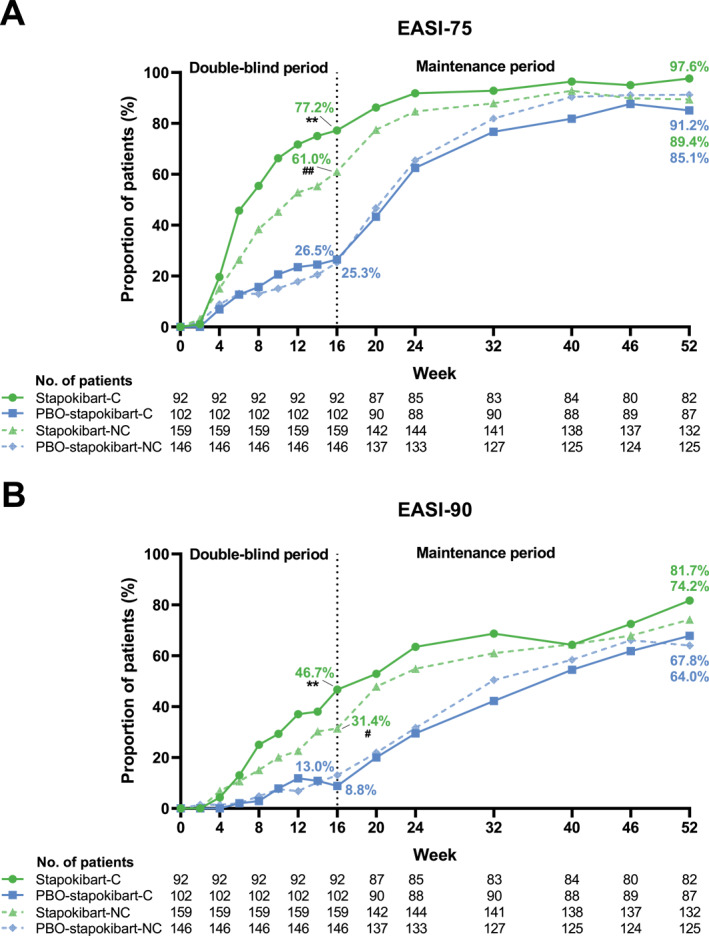
EASI‐75 (A) and EASI‐90 (B) response rates in patients with and without type 2 comorbidities during weeks 0–52. C, comorbid; EASI, Eczema Area and Severity Index; NC, non‐comorbid; PBO, placebo. **, *p* < 0.0001 compared with the placebo group in comorbid patients at week 16. ^##^, *p* < 0.0001; ^#^, *p* < 0.001 compared with the placebo group in non‐comorbid patients at week 16.

**FIGURE 2:**
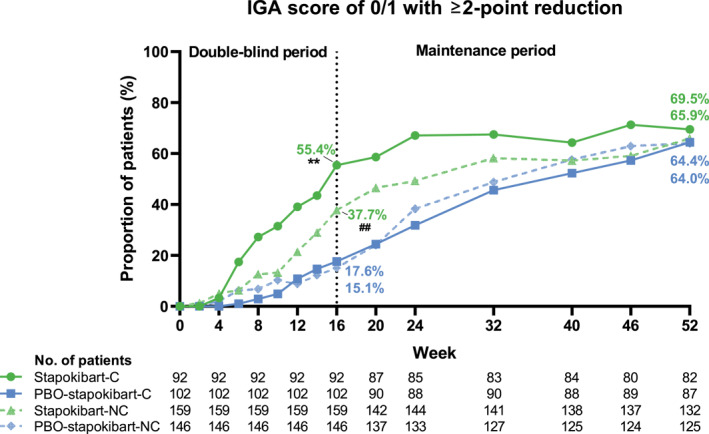
IGA 0/1 response rate in patients with and without type 2 comorbidities during weeks 0–52. C, comorbid; IGA, Investigator's Global Assessment score; NC, non‐comorbid; PBO, placebo. **, *p* < 0.0001 compared with the placebo group in comorbid patients at week 16. ^##^, *p* < 0.0001 compared with the placebo group in non‐comorbid patients at week 16.

At week 16, stapokibart demonstrated superior efficacy over placebo in proportion of EASI‐75, EASI‐90, and IGA 0/1 in patients with and without type 2 comorbidities. EASI‐75 was achieved in 77.2% (71/92) versus 26.5% (27/102) (*p* < 0.0001) of comorbid patients and 61.0% (97/159) versus 25.3% (37/146) (*p* < 0.0001) of non‐comorbid patients, respectively (Figure 1A). EASI‐90 response rate showed a similar trend, reaching 46.7% (43/92) versus 8.8% (9/102) (*p* < 0.0001) in comorbid patients and 31.4% (50/159) versus 13.0% (19/146) (*p* = 0.0001) in non‐comorbid patients, respectively (Figure 1B). Similarly, IGA 0/1 was achieved in 55.4% (51/92) versus 17.6% (18/102) (*p* < 0.0001) of comorbid patients and 37.7% (60/159) versus 15.1% (22/146) (*p* < 0.0001) of non‐comorbid patients, respectively (Figure 2).

**FIGURE 3:**
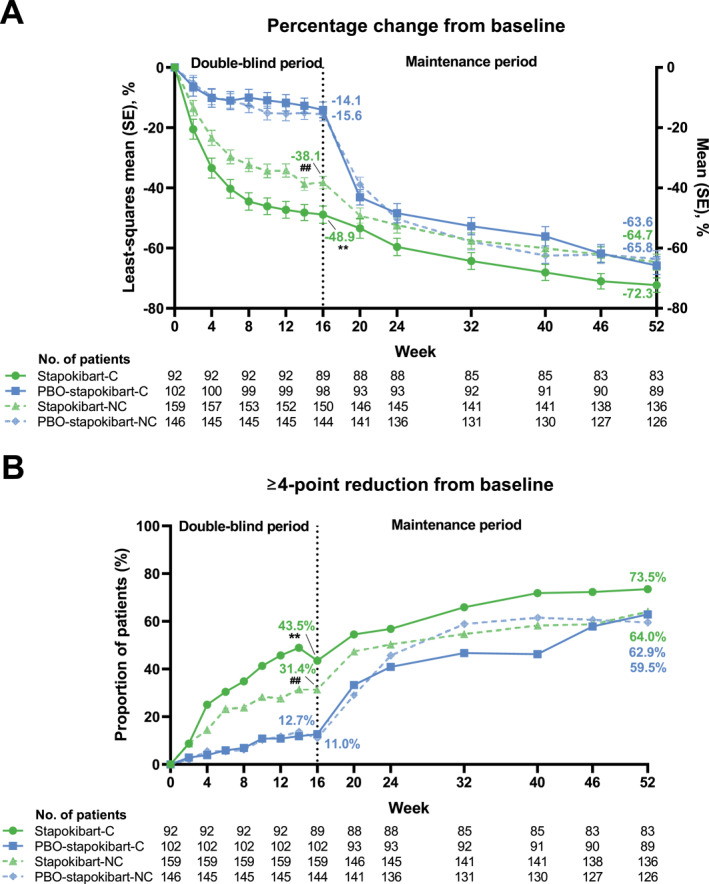
Improvement in weekly average of daily peak pruritus Numerical Rating Scale score in patients with and without type 2 comorbidities during weeks 0–52. (A) Percentage changes from baseline. (B) The proportion of patients achieving ≥ 4‐point reduction from baseline. C, comorbid; NC, non‐comorbid; PBO, placebo SE, standard error; **, *p* < 0.0001 compared with the placebo group in comorbid patients at week 16. ^##^, *p* < 0.0001 compared with the placebo group in non‐comorbid patients at week 16.

Stapokibart also achieved significantly greater pruritus relief compared with placebo at week 16. Percentage change from baseline in weekly average of daily PP‐NRS score was −48.9% versus −14.1% (least‐squares mean; *p* < 0.0001) and −38.1% versus −15.6% (*p* < 0.0001) in comorbid and non‐comorbid patients, respectively (Figure 3A). In addition, a significantly higher proportion of stapokibart‐treated patients versus placebo‐treated patients, with (43.5% [40/92] vs. 12.7% [13/102], *p* < 0.0001) or without (31.4% [50/159] vs. 11.0% [16/146], *p* < 0.0001) type 2 comorbidities, achieved PP‐NRS4 (Figure 3B).

In percentage change from baseline in weekly average of daily PP‐NRS score, the between‐group difference was significantly greater in the subgroup with type 2 comorbidities versus those without (−34.9% vs. −22.4%, interaction *p* = 0.0262). A borderline significant interaction was observed for EASI‐90 response rate (37.1% vs. 18.8%, interaction *p* = 0.0556), indicating a trend toward more pronounced improvement in the comorbid subgroup. For other efficacy outcomes, treatment effects did not show statistically significant interactions between subgroups (all interaction *p* > 0.1; Table 2).

**TABLE 2:** Efficacy outcomes at week 16 in patients with and without type 2 comorbidities.

Endpoints	Comorbid				Non‐comorbid				*P* for interaction
	Stapokibart (*n* = 92)	Placebo (*n* = 102)	Differences (95% CI)	*p* value	Stapokibart (*n* = 159)	Placebo (*n* = 146)	Differences (95% CI)	*p* value	
EASI‐75, *n* (%)	71 (77.2)	27 (26.5)	49.3 (35.8, 60.2)	< 0.0001	97 (61.0)	37 (25.3)	36.3 (25.5, 46.0)	< 0.0001	0.1465^a^
EASI‐90, *n* (%)	43 (46.7)	9 (8.8)	37.1 (24.8, 48.1)	< 0.0001	50 (31.4)	19 (13.0)	18.8 (9.5, 27.6)	0.0001	0.0556^a^
IGA 0/1, *n* (%)	51 (55.4)	18 (17.6)	35.1 (21.6, 46.9)	< 0.0001	60 (37.7)	22 (15.1)	23.9 (14.1, 33.1)	< 0.0001	0.4255^a^
Percentage change from baseline in weekly average of daily PP‐NRS score (%), LS mean (SE)	−48.9 (2.9)	−14.1 (2.7)	−34.9 (−42.7, −27.0)	< 0.0001	−38.1 (2.1)	−15.6 (2.2)	−22.4 (−28.5, −16.4)	< 0.0001	0.0262^b^
≥ 4‐point reduction from baseline in weekly average of daily PP‐NRS score, *n* (%)	40 (43.5)	13 (12.7)	30.2 (17.5, 41.8)	< 0.0001	50 (31.4)	16 (11.0)	19.8 (10.8, 28.4)	< 0.0001	0.3154^a^

Abbreviations: CI, confidence interval; EASI, eczema area and severity index; IGA, investigator's global assessment; LS, least‐squares; SE, standard error; PP‐NRS, peak pruritus numerical rating scale.

^a^ P for interaction was calculated using the logistic regression model, with the status of response (yes or no) as the dependent variable, and the baseline score, treatment, random strata (baseline IGA score of 3 vs. 4), subgroup (with or without type 2 comorbidities), and treatment‐by‐subgroup interaction as fixed effects.

^b^ P for interaction was calculated using the analysis of covariance model, with percentage change from baseline in weekly average of daily PP‐NRS score as the dependent variable, and the baseline PP‐NRS score, treatment, random strata (baseline IGA score of 3 vs. 4), subgroup (with or without type 2 comorbidities), and treatment‐by‐subgroup interaction as fixed effects.

Stapokibart‐treated patients demonstrated significantly greater reductions in pharmacodynamic biomarkers versus placebo during weeks 0–16, consistently observed in both comorbid and non‐comorbid groups (Supporting Information S1: Figure S1).

### 3.3 Efficacy Outcomes During Weeks 20–52

Following stapokibart treatment during the maintenance period, patients in all the four groups had further improvements in the response rates and daily PP‐NRS score continuously. At week 52, the response rates ranged from 85.1% to 97.6% for EASI‐75, 64.0%–81.7% for EASI‐90, 64.0%–69.5% for IGA 0/1, and 59.5%–73.5% for PP‐NRS4 (Figure 1, 2, 3). Percentage change from baseline in weekly average of daily PP‐NRS score ranged from −72.3% to −63.6% at week 52 (Figure 3A).

### 3.4 Safety Outcomes During Weeks 0–52

During 16‐week double‐blind treatment, the incidence of TEAEs was similar between stapokibart group and placebo group either with type 2 comorbidities (70.7% vs. 65.7%) or without (71.7% vs. 66.7%). The incidence of TEAEs was also comparable between comorbid and non‐comorbid patients exposed to stapokibart over weeks 0–52 (88.8% and 87.7%). The most common infections and infestations were COVID‐19, upper respiratory tract infection, and suspected COVID‐19. In patients receiving stapokibart treatment, conjunctivitis occurred in 5.9% patients with comorbidities and 5.0% patients without comorbidities over weeks 0–52. Severe TEAEs and SAEs were reported in 0.5% and 2.7% in patients with type 2 comorbidities and 4.3% and 6.6% in those without, respectively (Table 3).

**TABLE 3:** TEAEs in patients with and without type 2 comorbidities.

Events	Comorbid				Non‐comorbid			
	Stapokibart (W0‐16, *n* = 92)	Placebo (W0‐16, *n* = 102)	Stapokibart (W0‐52, *n* = 92)	Total stapokibart exposure (W0‐52, *n* = 187)	Stapokibart (W0‐16, *n* = 159)	Placebo (W0‐16, *n* = 147)	Stapokibart (W0‐52, *n* = 159)	Total stapokibart exposure (W0‐52, *n* = 302)
TEAEs	65 (70.7)	67 (65.7)	87 (94.6)	166 (88.8)	114 (71.7)	98 (66.7)	148 (93.1)	265 (87.7)
Drug‐related TEAEs	20 (21.7)	21 (20.6)	28 (30.4)	55 (29.4)	30 (18.9)	19 (12.9)	46 (28.9)	77 (25.5)
Severe TEAEs	0	0	0	1 (0.5)	2 (1.3)	1 (0.7)	8 (5.0)	13 (4.3)
SAEs	1 (1.1)	1 (1.0)	2 (2.2)	5 (2.7)	2 (1.3)	2 (1.4)	13 (8.2)	20 (6.6)
TEAEs leading to treatment discontinuation	0	0	0	2 (1.1)	2 (1.3)	1 (0.7)	7 (4.4)	10 (3.3)
TEAEs leading to death	0	0	0	0	0	0	0	0
TEAEs occurring in ≥ 5% of patients in ≥ 1 treatment group (systemic organ class/preferred term)								
Infections and infestations	41 (44.6)	34 (33.3)	76 (82.6)	138 (73.8)	64 (40.3)	66 (44.9)	117 (73.6)	191 (63.2)
COVID‐19	18 (19.6)	18 (17.6)	52 (56.5)	92 (49.2)	31 (19.5)	29 (19.7)	66 (41.5)	98 (32.5)
Upper respiratory tract infection	5 (5.4)	6 (5.9)	21 (22.8)	33 (17.6)	10 (6.3)	20 (13.6)	25 (15.7)	39 (12.9)
Suspected COVID‐19	5 (5.4)	4 (3.9)	12 (13.0)	16 (8.6)	8 (5.0)	9 (6.1)	23 (14.5)	40 (13.2)
Conjunctivitis	3 (3.3)	2 (2.0)	4 (4.3)	11 (5.9)	5 (3.1)	2 (1.4)	8 (5.0)	15 (5.0)
Folliculitis	2 (2.2)	4 (3.9)	4 (4.3)	7 (3.7)	4 (2.5)	8 (5.4)	6 (3.8)	15 (5.0)

*Note:* Data are *n* (%).

Abbreviations: SAE, serious adverse event; TEAE, treatment‐emergent adverse event; W, week.

## 4 Discussion

This post‐hoc analysis of CM310AD005 revealed that stapokibart resulted in significantly greater improvements in AD signs and pruritus symptom compared with placebo, both in patients with and without type 2 comorbidities. In addition, comorbid patients generally revealed greater treatment effect at week 16, as observed by EASI‐90 response rate and percentage change in weekly average of daily PP‐NRS score. The safety profile was generally comparable between the two subgroups.

During the double‐blind treatment period, in subgroups with and without type 2 comorbidities, stapokibart‐treated patients achieved significantly greater improvements compared with placebo‐treated patients, and the benefit was more pronounced in the subgroup with type 2 comorbidities at week 16. The greater effect size observed in this subgroup may be attributed to a more pronounced Th2‐driven immune phenotype, which is consistent with the mode of action of stapokibart. Serum TARC and total IgE are common type 2 inflammation markers, whereas serum LDH is related to general inflammation. In our study, patients with type 2 comorbidities had remarkably higher baseline serum total IgE levels than those without. Decreases in pharmacodynamic biomarkers during stapokibart treatment paralleled improvements in AD signs and symptoms. Specifically, during weeks 0–16 of stapokibart treatment, comorbid patients exhibited a slightly greater percentage reduction in TARC levels comorbid patients compared with non‐comorbid patients. This finding was consistent with longitudinal clinical studies of AD patients receiving systemic treatment, such as dupilumab, which demonstrated that TARC reduction was correlated with clinical improvement [17].

During the subsequent maintenance period, patients in all four groups achieved sustained improvements in the weekly average of daily PP‐NRS score and response rates including EASI‐75, EASI‐90, IGA 0/1, and PP‐NRS4. The improvements were similar at week 52, except for slight superiority in patients in the stapokibart group with type 2 comorbidities. Meanwhile, the presence of type 2 comorbidities did not seem to apparently increase the need for concomitant topical or systemic medications for AD. The efficacy data was consistent in the overall study population [15]. These findings were supported by prior analyses from clinical studies of dupilumab (anti‐IL‐4Rα) and lebrikizumab (anti‐IL‐13), both demonstrating significant improvements in AD signs and symptoms regardless of type 2 comorbidities [18, 19, 20, 21]. Analysis of the LIBERTY AD trials further confirmed consistent efficacy in adults and adolescents with AD with or without AR [18, 19]. This was consistent with a real‐life study involving adolescents and adults with AD, which showed no significant difference in efficacy and safety of dupilumab irrespective of comorbidities [20]. Similarly, pooled phase 3 trial data for lebrikizumab was generally equally efficacious in adults and adolescents with AD regardless of atopic comorbidities [21]. In contrast, tralokinumab, another anti‐IL‐13 antibody, exhibited superior treatment response in a subgroup of early‐onset AD with atopic comorbidities in a real‐world study [22]. Additional data from large‐scale studies in real‐world settings are warranted to fully elucidate the influence of atopic comorbidities on treatment response to biologics in patients with AD.

Stapokibart was generally well tolerated in adults with msAD either with or without type 2 comorbidities during 52‐week treatment. The incidence of any TEAEs was comparable, with conjunctivitis reported in around 5% of patients in both subgroups. Severe TEAEs and SAEs were low in all stapokibart‐treated patients, less than 3% in patients with type 2 comorbidities and less than 7% in those without, respectively. Furthermore, treatment discontinuation was low, 1.1% and 3.3% in the two subgroups, respectively. In other words, coexisting type 2 comorbidities did not lead to increased safety concern during long‐term stapokibart treatment. The overall safety was in line with the known safety profile of stapokibart [16].

This work has several limitations. First, it was a post‐hoc analysis and all *p* values were nominal. Second, the randomization was not stratified according to type 2 comorbidities. Third, the subgroups were classified according to comorbid conditions at baseline, which did not preclude the development of type 2 comorbidities subsequently. Finally, the outcomes related to asthma, AR, or other type 2 comorbidities were not analyzed. Stapokibart is under investigation for the treatment of asthma (NCT05761028), perennial allergic rhinitis (NCT06525597), and chronic obstructive pulmonary disease (NCT06547333). Future prospective studies will further elucidate the therapeutic potential of stapokibart in patients with multiple type 2 inflammatory diseases.

## 5 Conclusion

Stapokibart was effective and safe in adults with msAD both with and without type 2 comorbidities following long‐term treatment. Moreover, patients with type 2 comorbidities tend to achieve greater improvements with stapokibart treatment.

## Author Contributions

**Yan Zhao:** conceptualization, investigation, writing – original draft, writing – review and editing, resources. **Litao Zhang:** investigation, writing – review and editing, resources. **Liming Wu:** investigation, writing – review and editing, resources. **Bin Yang:** investigation, writing – review and editing, resources. **Jinyan Wang:** investigation, writing – review and editing, resources. **Yumei Li:** investigation, writing – review and editing, resources. **Qingchun Diao:** investigation, writing – review and editing, resources. **Jingyi Li:** investigation, writing – review and editing, resources. **Qing Sun:** investigation, writing – review and editing, resources. **Xiaohong Zhu:** investigation, writing – review and editing, resources. **Xiaoyong Man:** investigation, writing – review and editing, resources. **Lihua Wang:** investigation, writing – review and editing, resources. **Yanyan Feng:** investigation, writing – review and editing, resources. **Tao Cai:** investigation, writing –review and editing, resources. **Huiming Zeng:** investigation, writing – review and editing, resources. **Linfeng Li:** investigation, writing – review and editing, resources. **Jianyun Lu:** investigation, writing – review and editing, resources. **Hong Ren:** investigation, writing – review and editing, resources. **Fuqiu Li:** investigation, writing – review and editing, resources. **Qianjin Lu:** investigation, writing – review and editing, resources. **Xiaohua Tao:** investigation, writing – review and editing, resources. **Rong Xiao:** investigation, writing – review and editing, resources. **Chao Ji:** investigation, writing – review and editing, resources. **Wenjie Zhao:** methodology, writing – review and editing, data curation. **Wei Chu:** writing – review and editing, formal analysis, data curation. **Bo Chen:** conceptualization, methodology, writing – review and editing, supervision. **Jianzhong Zhang:** conceptualization, investigation, writing – review and editing, methodology, supervision, resources.

## Funding

This study was supported by Keymed Biosciences (Chengdu) Co. Ltd.

## Ethics Statement

This trial adhered to the ethical standards of the Declaration of Helsinki and followed the Good Clinical Practice regulations established by the National Medical Products Administration. The research protocol, including any modifications, received approval from the ethics committee all involved sites.

## Consent

Written informed consent was obtained from each participant prior to any study procedure.

## Conflicts of Interest

Wenjie Zhao and Wei Chu are employees of Keymed Biosciences (Chengdu) Co. Ltd. Bo Chen is a shareholder of Keymed Biosciences (Chengdu) Co. Ltd. All other authors declare no conflicts of interest.

## Supporting information


Supporting Information S1

## Data Availability

Data access could be provided by the corresponding author upon reasonable request.
