# Supplementary material for: Efficacy and Safety of Stapokibart in Adults With Moderate‐to‐Severe Atopic Dermatitis With and Without Type 2 Comorbidities: A Post Hoc Analysis of a Phase 3 Trial
Source: Clin Transl Allergy. 2025 Nov 19;15(11):e70121. doi: 10.1002/clt2.70121 (PMC12628279; doi:10.1002/clt2.70121)
Supplement: Supplementary file 1 — Supporting Information S1 [file CLT2-15-e70121-s001.docx]

**Supplementary Material**

**Figure S1.** Percentage change from baseline in pharmacodynamic biomarkers in patients with and without type 2 comorbidities during weeks 0-16. (A) Serum thymus and activation-regulated chemokine (TARC). (B) Serum total IgE. (C) Lactate dehydrogenase (LDH). Data are presented as median and interquartile range. C, comorbid; NC, non-comorbid; PBO, placebo.

**TABLE S1** Overview of concomitant medications for atopic dermatitis

| Concomitant medications | Comorbid | | | | Non-comorbid | | | |
| --- | --- | --- | --- | --- | --- | --- | --- | --- |
|  | Stapokibart (W0-16, n=92) | Placebo (W0–16, n=102) | Stapokibart (W16-60, n=89) | Placebo-stapokibart (W16-60, n=95) | Stapokibart (W0-16, n=159) | Placebo (W0-16, n= 147) | Stapokibart (W16-60, n=148) | Placebo-stapokibart (W16-60, n=143) |
| Topical corticosteroids | 4 (4.3) | 27 (26.5) | 12 (13.5) | 33 (34.7) | 6 (3.8) | 33 (22.4) | 14 (9.5) | 47 (32.9) |
| Topical calcineurin inhibitors | 2 (2.2) | 12 (11.8) | 8 (9.0) | 19 (20.0) | 9 (5.7) | 16 (10.9) | 13 (8.8) | 23 (16.1) |
| Systemic glucocorticoids | 0 | 1 (1.0) | 0 | 1 (1.1) | 1 (0.6) | 1 (0.7) | 0 | 0 |
| Janus kinase inhibitors | 0 | 1 (1.0) | 0 | 0 | 0 | 0 | 1 (0.7) | 1 (0.7) |
| Biologics | 0 | 1 (1.0) | 0 | 0 | 0 | 0 | 0 | 0 |

Data are n (%).
